# The sense of smell, its signalling pathways, and the dichotomy of cilia and microvilli in olfactory sensory cells

**DOI:** 10.1186/1471-2202-8-S3-S1

**Published:** 2007-09-18

**Authors:** Rebecca Elsaesser, Jacques Paysan

**Affiliations:** 1 Johns Hopkins University School of Medicine, 725 N. Wolfe St., 408 WBSB, Baltimore, MD 21205, USA; 2 Technical University of Darmstadt, Institute of Zoology, Schnittspahnstrasse 3, D-64287 Darmstadt, Germany

## Abstract

Smell is often regarded as an ancillary perception in primates, who seem so dominated by their sense of vision. In this paper, we will portray some aspects of the significance of olfaction to human life and speculate on what evolutionary factors contribute to keeping it alive. We then outline the functional architecture of olfactory sensory neurons and their signal transduction pathways, which are the primary detectors that render olfactory perception possible. Throughout the phylogenetic tree, olfactory neurons, at their apical tip, are either decorated with cilia or with microvilli. The significance of this dichotomy is unknown. It is generally assumed that mammalian olfactory neurons are of the ciliary type only. The existance of so-called *olfactory microvillar cells *in mammals, however, is well documented, but their nature remains unclear and their function orphaned. This paper discusses the possibility, that in the main olfactory epithelium of mammals ciliated and microvillar sensory cells exist concurrently. We review evidence related to this hypothesis and ask, what function olfactory microvillar cells might have and what signalling mechanisms they use.

## The "scentimental" nose

Wine experts occasionally identify a broad variety of aromas such as »bacon fat, pain grillé, black raspberries, cassis, white flowers, and Provençal olives« when they taste, for example, a vintage of Côte Rôtie [1]. Even an expert's nose, however, can get surprisingly confused in the presence of misleading visual cues. In a noteworthy study performed at the University of Bordeaux, Gil Morrot and collaborators asked 54 undergraduate students from the Faculty of Oenology to compare a glass of white Bordeaux wine (containing Sémillon and Sauvignon grapes) to a glass of exactly the same wine, which had been coloured by a mixture of red anthocyanins. Although the added anthocyanins had no perceptible taste or odour, the subjects identified remarkably different sets of aromas in both glasses [2]. Despite this surprising lack of discriminatory skills, it seems our sense of smell has other proficiencies, such as a remarkable power of attributing emotional qualities to objects that we see. The attributive nature of the sense of smell is illustrated by the fact that we need so-called descriptors to communicate odours [3]. What alternatives do we have but to portray an olfactory perception as »black raspberries-like«, or »Provençal olives-like«? None of these scents can be addressed directly, without reference to its respective source [4]. Other perceptual qualities can often be named independently. We can speak, for example, of »red« and »sweet« without employing metaphors and similes such as »anthocyanin-like« or »sucrose-like«. Odours, on the other hand, can affect our emotional state profoundly [5-7]. A gustatory stimulus, such as »too salty«, effectively keeps us from drinking sea water, but compared to bad odours, the aversion evoked by the taste of salt is less affective. This is vividly illustrated by an authentic report by the early Gabriel Garcia Marquez, who describes the fate of a shipwrecked sailor who drifts on his raft through the Caribbean sea. After four days of suffering the agonies of thirst, he decides to drink salt water. "This water does not satisfy your thirst", the salvaged sailor later testifies, "but it refreshes" [8]. Apparently, the repulsion evoked by the salty taste is based on reflex and rational thinking rather than on emotional disgust. By the same token, sweetness alone will rarely make us long for artificial sweeteners and it is unlikely to evoke a similar savouriness as the scent of freshly baked bread. In contrast, the repulsiveness of the nauseating stench of rotting meat is so effective that the use of this smell was suggested as a powerful stimulus for therapeutical conditioning of sexual offenders [9,10].

It seems that an important role of the human sense of smell is adding emotional qualities to situations and objects that we see, and not so much to making an essential contribution to finding or identifying things. Of course we can recognize typical odours even with our eyes shut, but the scent of ripe bananas, for example, rather influences our appetite than being necessary to distinguish bananas from pineapples. This observation raises questions about the biological purpose of the olfactory sense in a visually dominated species, such as man. What is so important about watering our mouth that it exerts sufficient selection pressure to sustain several hundreds of functional olfactory receptor genes through evolution? Why do we even maintain the ability to regenerate olfactory neurons from adult stem cells, just to make sure that our sense of smell stays alive as long as we do?

The functional anatomy of the mammalian nose and its close contact to the oral cavity point to possible answers to these questions. Odour molecules approach the olfactory epithelium along two principle routes: When we sniff or breathe with our mouth closed, the inhaled air enters the lumen of the nasal cavity through external nostrils. Food almost touches our nares before it enters the oral cavity, and it is almost impossible not to stick our nose into a glass of wine that we taste. When we chew, we break down the food into a mush from which favours exhale. These volatile odour molecules then travel up the retro-nasal passage and reach the olfactory epithelium through internal nostrils, which connect the pharynx to the nasal cavity [11]. The phylogenetic emergence of these internal nostrils (or choanae) was an important step in the adaptation of the olfactory system to air breathing [12]. It now enables us to employ olfactory perception in the assessment of nutrients at every level of uptake. We can now even evaluate compounds in our food that are made accessible only by mechanical exposure. This close involvement of the olfactory system in the analysis of food along with the attributive nature of olfactory perception allow us to employ the sense of smell in aquiring refined food preferences. In this respect, humans are uniquely flexible. There might be some inherently aversive odours (such as the smell of rotting meat), but depending on trends and cultural background, we can enjoy almost everything, from fried locusts spiced with salt and lime (as served in the Mexican region of Oaxaca) to live oysters (which are perceived as a delicacy in a lot of wealthy Western societies). We can even "learn to enjoy things that we should not enjoy" [13]. Used as flavoring agents, bitter compounds, including plant-derived phenols and polyphenols, flavonoids, catechins, and caffeine, enhance the sensory appeal of beverages and food, such as beer, coffee, chocolate, tea, and tonic water [14]. This flexibility effectively supports opening up new food resources. In this context, olfactory conditioning helps us to pass on approved food preferences to our infant offspring, while at the same time protecting them from exploring potentially noxious material while unattended (long before they understand the advise that we give). Cooking-ambitious parents often experience how cautious and how annoyingly conservative their children can be, when it comes to trying out unknown delicacies. The juveniles may well obstinately insist on fish sticks with mashed potatoes when served cod steaks cooked in an »interesting spice mixture of ginger, cloves, chilies, cilantro leaves, and lemon juice« [15]. Training pays out, however, and we can learn to expand our pool of nutrients to include surprisingly variable ingredients.

For free living rats, the importance of social learning of food preferences has also been demonstrated [16], and olfaction certainly plays an important role in this process [17]. Mice prefer sweet over bitter, even if the sweet perception is triggered erroneously by a bitter compound [18]. Humans, in contrast, are capable of expanding their menu well beyond sweet and non-bitter aliments, and they seem to do so by prioritizing hedonic odour qualities over gustatory perception, and by dynamically adapting their food preferences to changing age, resources, and trends.

The production of aromas and scents has long been an important economical factor [19] and an interesting perspective for biotechnological fabrication [20]. Olfactory signals can act as mediator of social interactions [21] and the potential of olfactory cues as marketing tools in social and economical context has long been recognized [22-24]. Nevertheless, empirical research in the field of how we perceive odours is still fairly new, although we know more today about our brain's strategy in computing odour perception [25-28] than we did only a few years ago. Innovative techniques such as genetic tracing of neural circuits [29-31] and functional magnetic resonance imaging (fMRI) [32-36] produced exciting new results. It turns out, that, even in insects, coding and representation of odours is highly complex and dynamic [27,28,37-39], but significant progress is made, and, for example, we now begin to understand why a typical white wine tastes like a typical red wine, just because we added a tiny pinch of neutral anthocyanins. In a striking fMRI study, Jay Gottfried and Raymond Dolan recently found evidence of why "the nose smells what the eye sees". Their data indicate that the "human hippocampus mediates a reactivation of crossmodal semantic associations, even in the absence of explicit memory processing" [32,33]. Apparently, »black raspberries and Provençal olives« are imprinted in our brain, while the nose just pulls the trigger to unfold them in our mind.

## Olfactory sensory neurons

The initial event in all these fascinating aspects of odour sensation is the binding of odorant molecules [40] to olfactory receptor proteins [41,42]. The released binding energy is transduced by a complex chain of molecular interactions into electrochemical membrane potentials, which dynamically modulate neuronal odour representation [19,43-56]. How do these primary olfactory processes work and where do they occur?

A crux of this matter are the bipolar chemosensory neurons in the olfactory epithelium of the nose. Olfactory neurons are based at the front line between the brain and the odour-containing air that we breathe. Their surface membrane can be divided into two major spatial compartments, which are separated by a belt of tight junctional proteins [57]. The basolateral surface membrane lies well protected inside of the olfactory epithelial tissue. It covers large part of the apical dendrite, the cell body, and an unbranched axon that projects to the brain. On the other hand, there is an apical surface membrane compartment, the size of which is comparable to that of the basal compartment. It is located externally, directly exposed to the air, and attached to the rest of the cell only through the nexus of a thin apical dendrite, which reaches into the lumen of the nasal cavity with its terminal knob. The apical surface membrane is embedded into mucus and airway surface fluid. These represent ionic and biochemical compartments, which are important for olfactory signal transduction [58,59], as well as for innate defense of all airway epithelial surfaces [60]. Disturbances of the composition of these fluids, e.g. by mutation of ion transport systems, can cause severe pathologies, including cystic fibrosis [61].

Through their apical membrane compartment, olfactory sensory neurons contact the ambient air. At this surface, they collect information about volatile odour molecules, and here odours are transduced into neuronal signals, which the brain can read and analyse [62-64]. The molecular machinery that mediates olfactory signal transduction has been thoroughly characterized. Briefly, volatile odour molecules bind to odorant receptors [65] and trigger the activation of G-proteins (Gα_olf_) [47]. This in turn stimulates adenylyl cyclase (type III) activity [66], and the resulting rise of intracellular adenosine 3',5'-cyclic monophosphate (cAMP) opens cyclic nucleotide-gated (CNG) cation channels [67]. The elicited influx of Ca^2+ ^from the extracellular space depolarizes the ciliary membrane and prompts a secondary opening of Ca^2+^-gated chloride channels. These are responsible for large part of the depolarizing current across the olfactory ciliary membrane [68,69]. The depolarizing nature of the induced chloride current is based on an unusually high intracellular chloride concentration in the cytoplasm and cilia of olfactory sensory neurons, which is replenished by sodium-potassium-chloride co-transporters (NKCC-1) in the basolateral surface membrane compartment [68,69]. Odour-induced signals are thought to be terminated as a result of multiple factors and mechanisms [70], although recent evidence indicates that the dwell-time of the receptor-odorant complex might be too small for some inactivation processes to occur under physiological conditions [71].

Beside this main stream model of olfactory signal transduction survived a scuff resistant hypothesis, proposing InsP_3 _as an alternative second messenger in olfaction [50,72,73]. The possibility of a supplementary signalling pathway in olfactory neurons was initially raised by a study, in which the authors found that in contrast to most odours, some potent odorants failed to induce cAMP when applied to isolated olfactory cilia in biochemical assays [74]. It was later found that these "non cAMP odours" induced InsP_3 _instead [75,76], and that odorant receptors coupled to either cAMP or InsP_3 _when they were functionally expressed in insect cells, depending on the receptor's specificity for either "cAMP-" or "IP3-odours" [77]. Despite these findings, the resulting speculation around a possible duality of signalling pathways in olfactory neurons remained controversial [49,78]. The goose of InsP_3 _as an alternative second messenger in olfactory neurons got cooked, when mutant mice that lacked functional expression of Gα_olf _[79], adenylyl cyclase (type III) [80], and cyclic nucleotide-gated ion channels [81] came out anosmic for both cAMP- and InsP_3_-odours. Consequently, many researchers began to accept that cAMP might be the sole excitatory second messenger in olfaction [82-84]. The evidence linking phosphoinositide-related signalling to the mammalian olfactory epithelium, however, remained orphaned.

## Cilia and microvilli

The apical surface membrane of olfactory sensory neurons is a platform that links the physical world of odour molecules to the mental world of odour perception. At this interface, the physiochemical properties of volatile compounds are transduced into the electrochemical membrane potentials, which modulate the information from which the brain constructs, for example, the aromas of »black raspberries« and »pain grillé«. The apical surface membrane compartment therefore represents a most important cell organelle. It is significantly enlarged by cilia or microvilli, which emerge from the terminal knob of the apical dendrite to protrude into the mucus [85]. What is the biological significance of this enlargement? Why do some sensory neurons sustain this extension with cilia, while others possess microvilli or both?

A requirement for high sensitivity is often regarded as a sufficiently meaningful biological reason for the enlargement of the surface area of sensory membranes. In photoreceptor cells, this argument intuitively makes sense. A photon on its path through a photoreceptor cell is either captured by a photosensitive rhodopsin molecule or the information it carries will be lost by absorption in non sensory material. Therefore, photoreceptor cells increase their quantum efficiency by forming multilayered stacks of membranes that are tightly packed with photosensitive rhodopsin molecules. This functional cytoarchitecture can likewise be observed in vertebrate [86] and invertebrate eyes [87], where stacks of membrane discs or rhabdomeric microvilli ensure high quantum efficiency of each photosensitive cell. In the case of olfactory sensory neurons, however, the situation is slightly different. Compared to photons, odour molecules are stable and could theoretically be trapped and retained in a lipophilic environment until receptor activation has occurred. Furthermore, the physiological purpose of increasing sensitivity is also not as obvious as a first glance might suggest. It has been reported that olfactory neurons are sufficiently sensitive to detect even single odour molecules [88], but the biological significance of this finding was challenged [49,89,90]. Extraordinary sensitivity has undoubtedly been observed in the olfactory system of moths [91], but since only a few mammals fly around in the dark with a need to smell their food or mating partner from miles away, it remains unclear how solitary odour molecules could carry meaningful information and why they should be detected at all. Quantal sensitivity of olfactory neurons, similar to that observed in photoreceptor cells [92,93], could also provide confusing sensory input to the brain. Olfactory neurons express only one or a few olfactory receptors [94,95] and many olfactory receptors are broadly tuned to overlapping sets of qualitatively distinct odour molecules [62-64]. Thus, two identical solitary odour molecules could trigger different sensory input into the olfactory system, depending on which receptor they hit by chance. Such an arrangement does not appear like a plausible approach to ensure reliable and reproducible sensory input, particularly at extremely low levels of odorant concentration. Accordingly, in contrast to phototransduction, olfactory signal transduction lacks amplification at one of its very basic levels, namely that of receptor-G-protein-activation. The life-time of the receptor-odorant complex was found to be so short, that the complex might often dissociate even before a single G-protein was activated. Consequently, signal amplification by one active receptor triggering multiple downstream G-protein/effector enzyme molecules seems unplausible [71].

Alternative explanations for the observed enlargement of the apical membrane compartment of olfactory neurons include the outstanding ability of these cells to provide information that allows the detection of even minute differences between odour concentrations, or, equivalently, differential affinities of odour molecules to distinct receptor populations.

Even humans are capable of extracting spatial information from smell [96-98]. Dogs just need five footsteps to determine the direction of an odour trail [99] and a rat can determine the location of an odour source by stereo-localisation in a single sniff [100]. To enable such impressing performances, it will most likely be necessary to maximize the dynamic sensitivity of olfactory neurons. The dynamic sensitivity represents the ability of a signalling system to respond to a small change of the input signal by modulating the output signal in a statistically significant manner. The dynamic sensitivity of an olfactory neuron is directly influenced by the size of its apical membrane surface compartment, because the latter is correlated to the number of signalling proteins (i.e. receptor molecules, G-proteins, and ion channels) and thus to the total number of elementary responses that can simultaneously be generated in each cell.

The requirements to a high performance odorant detector can be illustrated by comparing it to a light detecting device, such as a charge-coupled device (CCD) camera. Here, each photosensitive element (analogous to an olfactory neuron in the nose) is characterized by its background noise, its quantum efficiency, *and *its full well capacity [101]. In a high performance CCD camera, noise reduction is achieved by cooling the sensor to low temperatures. Quantum efficiency of a photon detector describes the ratio between the number of photons registered by the detector to the number of photons entering the device. Lastly, the full well capacity of the photon detector describes the total number of photons that can be registered within a single readout cycle. To improve the dynamic sensitivity of any charge-coupled device, it is necessary to combine high quantum efficiency with a large full well capacity. If the ratio between full well capacity and quantum efficiency of the detector is low, the slope of its response curve will be shallow. This, in turn, makes it difficult to reliably detect minute differences in signal amplitude, particularly when background noise is high. Therefore, high performance light detectors require the optimization of all three parameters combined: low background noise, high quantum sensitivity, and large full well capacity.

For olfactory sensory neurons, similar requirements might apply. In the olfactory sytem, noise reduction is a multi level process [102]. It begins with a nonlinear signal amplification by Ca^2+^-activated chloride currents [103,104], continues by the convergence of dozens of cilia onto the terminal knob of the sensory neuron's apical dendrite, and is further sophisticated by the convergence of hundreds of olfactory neurons on a single glomerulus in the olfactory bulb [105-107]. This multilevel integration with the possibility of adjusting thresholds at each level enables effective filtering of background noise, which has been observed as an intrinsic feature of olfactory signal transduction [102,108-111]. To represent high performance detectors for chemical constituents of the air, olfactory neurons might also have to improve quantum efficiency and full well capacity by enlarging their apical membrane compartment. At a given odour concentration and affinity of the odorant to its receptor, the quantum efficiency of the olfactory neuron as a whole will directly depend on the total number of receptors present. The same holds true for the neuron's "full well capacity", which represents its capacity to register odorant-to-receptor-docking-events within a single readout cycle (which in this case might be equivalent to the duration of a sniff). Since there is a limit to packaging density of receptors and other signaling molecules within the surface membrane, these parameters will also depend on the total apical surface membrane area. Olfactory neurons therefore enlarge this area by the folding the apical membrane into cilia and/or microvilli [85]. Interestingly, it seems that olfactory neurons further improve their physiological performance by additional measures. As mentioned above, the receptor-odorant dwell-time is very short, enabling repeated binding of odorant molecules to the same receptor. Furthermore, the quantal responses of olfactory neurons are very small. At the same time, the density of receptors in the ciliary membrane is so high that their response domains overlap, which causes non linear summation of the odour-induced unitary responses [71]. Since linearity is neither necessary for detecting differential activation patterns (i.e. specific odours), nor for sensing relative differences in odour concentration, this strategy seems to improve the dynamic resolution of each olfactory neuron over a broad range of odour concentration levels and thus perfectly serves to the needs of a biological high performance chemodetector.

### Ciliary and microvillar (rhabdomeric) photoreceptor cells

For animal photoreceptor cells, two types of membrane enlargement have been recognized. Rhabdomeric photoreceptor cells, as they occur in the compound eye of arthropods, carry microvilli, while the rods and cones of the mammalian retina and the light sensitive cells of the pineal organ are of a ciliary type. The evolutionary origin of both cell types was unclear [112], until Detlev Arendt and his collaborators found a polychaete marine worm, *Platynereis dumerilii*, which possesses both, rhabdomeric and ciliary photoreceptors [113]. On the basis of these findings, the authors proposed an attractive model for the evolution of metazoan photoreceptor cells. Accordingly, rhabdomeric and ciliary photoreceptors have emerged from a common ancestor that used an ancestral opsin for photodetection. The ancestral opsin gene then duplicated into the paralogs rhabdomeric opsin (r-opsin) and ciliary opsin (c-opsin), supporting the emergence of ciliated and rhabdomeric photoreceptor cells and their functional diversification. This dualism of light sensitive cells can also be observed within the mammalian retina. Melanopsin, the vertebrate ortholog of invertebrate r-opsin, is expressed in some light-sensitive retinal ganglion cells, which are involved in synchronizing the primary circadian pacemaker in the suprachiasmatic nucleus to the light-dark cycle [114].

Light transduction in invertebrates is mediated by an eye-specific phospholipase C (norpA), phosphatidylinositol 4,5-bisphosphate (PIP_2_), its downstream products InsP_3 _and diacylglycerol [115-117], and transient receptor potential (TRP and TRPL) channels [118,119]. Interestingly, the vertebrate ortholog (PLC β4) of norpA is also expressed in the mammalian retina [120] and melanopsin signalling has been linked to InsP_3 _in cultured *Xenopus *melanophores [121-124]. This raises the question, whether cells expressing r-opsin and its orthologs generally utilize phosphoinositides as second messengers. Although melanopsin-expressing retinal ganglion cells do neither appear to be normal retinal ganglion cells nor specialized rhabdomeric or microvillar sensory neurons [125,126], this evidence nevertheless points towards a possible phylogenetic link between rhabdomeric photoreceptor cells and phosphoinositide-mediated signalling processes. In contrast, ciliary photoreceptors prefer phosphodiesterase-mediated signal transduction with cGMP as a second messenger [121,122].

A functional reason for the possible allocation between microvilli and InsP_3 _signalling has been proposed by Klaus Lange [127]. He suggested that F-actin, which is a major constituent of the cytoskeleton of microvilli, represents an InsP_3_-sensitive, non vesicular Ca^2+^-store. Accordingly, a stimulation of phospholipase C will release the actin-binding proteins profilin and gelsolin from phospholipids, which in turn liberates Ca^2+ ^from F-actin. InsP_3 _stabilizes the active state of profilin/gelsolin. Furthermore, PIP_2_, a substrate of phospholipase C, is preferentially localised to raft-like lipid domains, which are typically observed in conjunction with microvilli [128,129]. This raises the possibility that, for principle reasons, signal transduction processes in cilia and microvilli preferentially utilize cyclic nucleotides and phosphoinositides as second messengers, respectively.

### Ciliated and microvillar olfactory neurons

In mammals, the apical cell surface of typical olfactory neurons is enlarged by a species-dependant number of cilia. In microvillar olfactory neurons the enlargement occurs by the folding of the membrane into microvilli. Both types of olfactory sensory neurons have been observed without perceivable phylogenetic boundaries throughout the clade of vertebrates. In her excellent and comprehensive review, Heather Eisthen pointed out that sharks, rays, and ratfish have only microvillar olfactory neurons, while lampreys, frogs, snakes, and turtles have only ciliated ones. In hagfish, bony fishes, and salamanders, both types of olfactory neurons can be observed next to each other, while in birds individual olfactory neurons simultaneously carry cilia and microvilli [85]. The situation in mammals is not entirely clear. Many researchers assume that only ciliated olfactory neurons are present, but the occurrence of microvillar olfactory neurons has also been proposed [130,131]. Surprisingly, this finding has never attracted much attention.

## Olfactory microvillar cells

**Figure 1:**
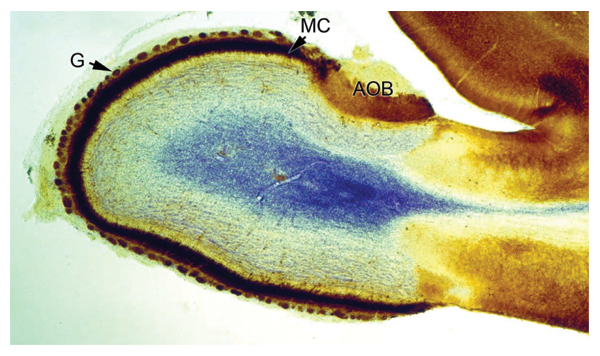
**Olfactory bulb**. Section through the olfactory bulb of a 16 days old rat brain. The tissue has been fixed and immunoperoxidase-stained with antibodies against GABAA-receptor_1-subunit (brown) as described elsewhere [157]. Nissl staining was performed to counter stain (blue). Clearly visible are the accessory olfactory bulb (AOB), to which chemosensory neurons from the vomeronasal organ project, the intensely labelled layer of mitral cells (MC), and the glomeruli (G), which represent the first relay station for sensory information transmitted from the nose to the brain (Jacques Paysan, unpublished).

**Figure 2:**
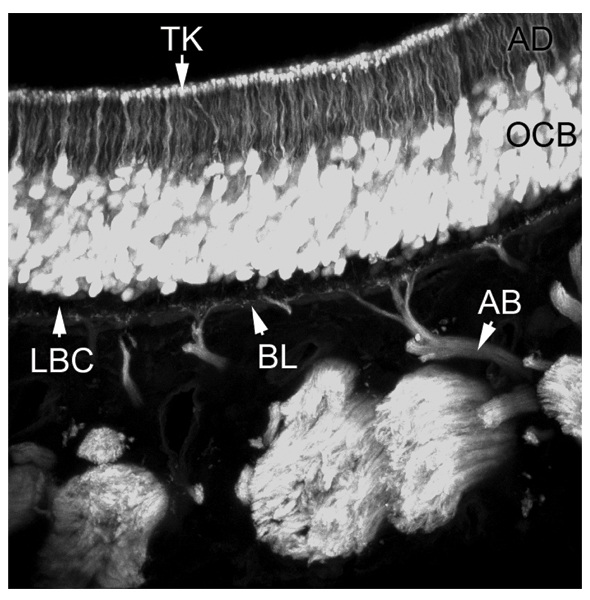
**Olfactory epithelium**. This image shows a vertical projection of a stack of confocal images taken from a transgenic mouse, in which green fluorescent protein (GFP) is expressed in all ciliated olfactory sensory neurons. GFP brightly labels the olfactory neuronal cell bodies (OCB), their apical dendrites (AD), and terminal knobs (TK). Staining does not extend into the sensory cilia, which remain invisible in this preparation. Basally to the olfactory neuronal cell bodies is the unstained layer of basal stem cells (LBC), from which degenerating neurons are constantly regenerated. The olfactory axons grow in bundles (AB) through the basal lamina (BL), and then fasciculate to form the tracts of the olfactory nerve, which projects into the brain. (Rebecca Elsaesser and Jacques Paysan, unpublished).

The olfactory epithelium of mammals consists of a relatively small number of cell types [132]. A monolayer of supporting or sustentacular cells separates the nasal cavity from the sensory neuroepithelium. This palisade-like monolayer covers a pseudostratified layer of bipolar sensory neurons, which basally project an axon to the olfactory bulb (Figure 1). Apically, the olfactory neurons contact the lumen of the nasal cavity with the terminal knob of their apical dendrite (Figure 2). Between the cell bodies of the chemosensory neurons and the basal lamina is a thin layer of so-called globose and horizontal basal cells, which contains a population of adult neuronal stem cells [133-136]. These stem cells are important to maintain function and histology of the olfactory epithelium, because based on their exposed situation in contact to ambient air, olfactory neurons are prone to damage and infection. Consequently, olfactory neurons have a limited lifetime before they are periodically replaced [137-141].

In addition to these three well established major olfactory epithelial cell types, i.e. ciliated olfactory sensory neurons, microvillous supporting or sustentacular cells, and basal cells, at least five classes of so-called olfactory microvillar cells exist [132]. To prevent confusion, Bert Menco and Edward Morrison suggested to systematically re-classify olfactory microvillar cells, and to combine all olfactory epithelial cells that have microvilli under the term "microvillous cells" [132]. The function of this peculiar type of cells is totally unknown.

**Figure 3:**
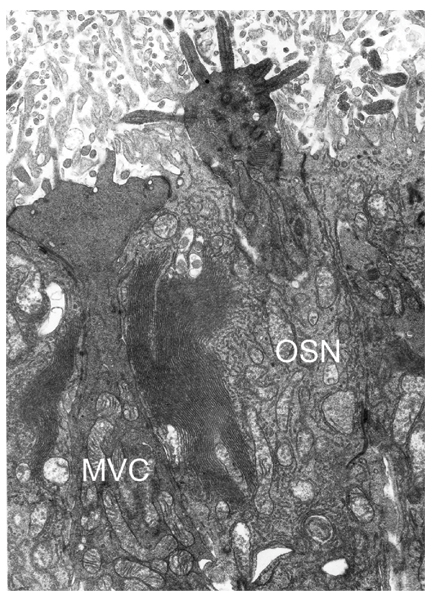
**Microvillar and ciliated olfactory sensory cells**. Electron micrograph showing the apical poles of an olfactory microvillar cell (MVC) and a typical olfactory sensory neuron (OSN). This image was generated in 1975 at the Centre de microscopie électronique (Claude Bernard University, Lyon 1, France) and kindly provided by François Jourdan. See [130] for details.

Some microvillous cells have been proposed to represent a second class of bipolar sensory neurons. Based on an electron microscopic study of the olfactory epithelium of rat, François Jourdan first made this suggestion in 1975 [130]. In his thesis, he described a microvillar type of bipolar olfactory cells (type B cells), the morphology of which, in his view, was that of a typical bipolar receptor neuron (Figure 3). Similar cells were later reexamined by David Moran, Carter Rowley, and Bruce Jafek [131], who also found evidence that supported the existence of a second morphologically distinct class of bipolar sensory neurons in the mammalian olfactory epithelium [142,143]. The authors injected horseradish peroxidase into the olfactory bulb of rat and detected retrograde labelling of olfactory epithelial microvillar cell bodies. They concluded that microvillar cells are connected by an axon to the olfactory bulb, and that the rat olfactory system will thus "need to be expanded to include two morphologically distinct classes of sensory receptors, ciliated olfactory receptors and microvillar cells" [143]. This hypothesis seemed plausible on the basis of the observation that ciliated and microvillar olfactory neurons occur in many other species throughout the clade of vertebrates [85,144]. However, the evidence that microvillar cells are neuronal remained controversial. When Virginia Carr and collaborators stained rat olfactory epithelia with monoclonal antibody 1A-6, they labelled microvillar cells that were not immunoreactive with SUS-1 antibodies (which is a marker for supporting cells). The 1A-6 immunoreactive cells, however, neither expressed olfactory marker protein (OMP), nor did they possess an identifiable axonal process. Since the cells also survived bulbectomy-induced degeneration of olfactory sensory neurons, the authors concluded that olfactory microvillar cells are of non-neuronal nature [145]. The absence of OMP in microvillar cells was confirmed by Edward Johnson and collaborators [146]. These authors also acknowledged some arguments that speak for a non-neuronal function of microvillar cells, but pointed out that the peroxidase backfill experiments [143] and the expression of Spot-35 protein [147] are in favour of positioning microvillar cells "in with known microvillar olfactory receptor cells of other vertebrates". They carefully concluded, that the function of the olfactory microvillar cell remains enigmatic [146].

Recently, we have examined the distribution of phosphoinositide-related signalling proteins in the mammalian olfactory epithelium and found that some elements of InsP_3_-mediated signal transduction were found exclusively in olfactory microvillar cells [19,148]. These cells that we call "Jourdan cells", were labelled with antibodies against phospholipase C beta-2 (PLC β2), type 3 InsP_3_-receptors (InsP_3_R-III), type 6 transient receptor potential channels (TRPC6), and other proteins, including neuronal marker protein MAP2b [148]. In contrast, no co-expression with OMP, cyclic nucleotide-gated channels, or adenylyl cyclase could be detected. When dissociated and exposed *in vitro *to depolarizing concentrations of potassium chloride, Jourdan cells reacted with a transient increase of their intracellular Ca^2+ ^concentration. A similar reaction was observed, when the cells were exposed to odours. They also possessed a basal axon-like protrusion, which raised the possibility that they in fact represent a second class of olfactory sensory neurons. In contrast to ciliated olfactory sensory neurons [149-151], however, microvillar cells did not degenerated upon bulbectomy. This has been interpreted as one argument for a non neuronal nature of olfactory microvillar cells [145,152].

Is there a duality of signalling pathways and sensory neurons in the mammalian olfactory epithelium, similar to the existence of ciliary and rhabdomeric photoreceptor cells? Unfortunately, it is too early to submit such an admittedly attractive proposal. At present, only a few arguments that can be brought in position to support any concept on what olfactory microvillar cells might do. First, these cells look like sensory neurons [130]. They show a strong bipolar asymmetry, with a distinctive array of microvilli protruding towards the nasal cavity at their apical pole, and an axon-like process, the distal extension of which often crosses the basal lamina and seems to join the submucosal tracts of the olfactory nerve [148]. This bipolar architecture of microvillar cells would at least be tailor made to enable the cells to collect signals at the olfactory mucosal surface and transmit this information across the epithelium. This view is supported by the localization of PLC β2 in the apical microvilli. PLC β2 is a G-protein-activated signal transduction enzyme and thus points towards the presence of yet unidentified upstream G-protein-coupled receptors. Moreover, the cells express neuronal marker protein MAP2b [148], Ca^2+^-binding protein SPOT-35 [153], and they are depolarized by high potassium chloride [148]. All of these findings are compatible with a role of olfactory sensory neurons, but none of them is a persuasive argument. To determine, whether or not olfactory microvillar cells in fact represent sensory neurons, it will be necessary to characterize them by electrophysiological recordings and to analyze their cell morphology and gene expression profile in greater detail.

Albeit their (un)neuronal nature, we have now found evidence that points towards a possible function of olfactory microvillar cells. As pointed out above, olfactory sensory neurons periodically die by apoptosis, being constantly regenerated from a distinct population of adult neuronal stem cells. To avoid unproportional growth or degeneration of the olfactory epithelial tissue throughout life, both processes – cell death and regeneration – must be precisely coordinated over many decades. It has been demonstrated by Donna Hansel, Betty Eipper, and Gabriele Ronnett, that among the factors, which contribute to this control, are amidated neuropeptides [154]. In the adult olfactory neuroepithelium, neuropeptide Y is released from an uncharacterized set of cells [155]. We were now able to show that the NPY-expressing cells are identical to the olfactory microvillar cells that we had previously characterized [156]. This finding raises the possibility, that olfactory microvillar cells link signals on the surface of the olfactory mucosa to proliferation and differentiation of olfactory stem cells at the basis of the epithelium. Whether or not olfactory microvillar cells indeed represent a second class of olfactory sensory neurons remains elusive. In analogy to melanopsin-expressing retinal ganglion cells, they could be derived from an ancient type of microvillar olfactory neurons, which in the course of evolution have aquired a new physiological function. These thoughts, however, are totally speculative. Our efforts to understand their biological significance have just begun. A first step must be to unravel their input specificity by identifying the G-protein-coupled receptors, which act upstream of PLC β2.

## Authors' contributions

JP outlined the manuscript and wrote the initial draft. RE significantly contributed to its contents, figures, and to finalizing the manuscript. Both authors read and approved the final manuscript.

## Competing interests

The authors have no competing interests.
